# Supplementary material for: SyNDI: synchronous network data integration framework
Source: BMC Bioinformatics. 2018 Nov 6;19:403. doi: 10.1186/s12859-018-2426-5 (PMC6219086; doi:10.1186/s12859-018-2426-5)
Supplement: Supplementary file 5 — Files of the example presented in Additional file 4. This additional file is a zip package that contains all files used in Additional file 4. Detailed descriptions of each of these files are presented in a README file included in this zip package. (GZ 30411 kb) [file 12859_2018_2426_MOESM5_ESM.gz › SyNDITools/Meme2Fimo/meme_4.9.0_4.tar.gz!/meme_4.9.0/website/html/canvas-test.html]

Canvas Test


## Canvas Logo Test

The purpose of this page is to provide a test for the canvas based logo functionality of meme.
As this function has caused us a lot of problems getting it right on different browsers this is intended to be a page
that users can take screenshots of to demonstrate problems.

| Test | Canvas Logo |
| --- | --- |
| IUPAC DNA codes; uniform background; no pseudocount; (ACGTMRWSYKVHDBN) |  |
| IUPAC AA codes; uniform background; no pseudocount;  (ACDEFGHIKL  MNPQRSTVWY  BZX) |  |
| DNA 'A' variable height; uniform background; |  |
| DNA 'C' variable height; uniform background; |  |
| DNA 'G' variable height; uniform background; |  |
| DNA 'T' variable height; uniform background; |  |
